# Functional antagonism of *TMPRSS2-ERG* splice variants in prostate cancer

**DOI:** 10.18632/genesandcancer.25

**Published:** 2014-07

**Authors:** Anshu Rastogi, Shyh-Han Tan, Ahmed A. Mohamed, Yongmei Chen, Ying Hu, Gyorgy Petrovics, Taduru Sreenath, Jacob Kagan, Sudhir Srivastava, David G. McLeod, Isabell A. Sesterhenn, Shiv Srivastava, Albert Dobi, Alagarsamy Srinivasan

**Affiliations:** ^1^ Center for Prostate Disease Research, Department of Surgery, Uniformed Services University of the Health Sciences, Bethesda, MD, USA;; ^2^ Cancer Biomarkers Research Group, Division of Cancer Prevention, National Cancer Institute, Bethesda, MD, USA;; ^3^ Urology Service, Department of Surgery, Walter Reed National Military Medical Center, Bethesda, MD, USA;; ^4^ The Joint Pathology Center; Silver Spring, MD, USA.

**Keywords:** *ERG*, Splice variants, Prostate cancer, Dominant negative, *C-MYC*

## Abstract

The fusion between *ERG* coding sequences and the *TMPRSS2* promoter is the most prevalent in prostate cancer (CaP). The presence of two main types of *TMPRSS2-ERG* fusion transcripts in CaP specimens, *Type I* and *Type II*, prompted us to hypothesize that the cumulative actions of different *ERG* variants may impact CaP development/progression. Using *TMPRSS2-ERG3* (*Type I*) and *TMPRSS2-ERG8* (*Type II*) expression vectors, we determined that the *TMPRSS2- ERG8* encoded protein is deficient in transcriptional regulation compared to *TMPRSS2-ERG3*. Co-transfection of vectors resulted in decreased transcriptional regulation compared to *TMPRSS2-ERG3* alone, suggesting transdominance of ERG8. Expression of exogenous ERG8 protein resulted in a decrease in endogenous ERG3 protein levels in *TMPRSS2-ERG* positive VCaP cells, with a concomitant decrease in C-MYC. Further, we showed a physical association between ERG3 and ERG8 in live cells by the bimolecular fluorescence complementation assay, providing a basis for the observed effects. Inhibitory effects of *TMPRSS2-ERG8* on *TMPRSS2- ERG3* were also corroborated by gene expression data from human prostate cancers, which showed a positive correlation between *C-MYC* expression and *TMPRSS2-ERG3/TMPRSS2- ERG8* ratio. We propose that an elevated *TMPRSS2-ERG3/TMPRSS2-ERG8* ratio results in elevated *C-MYC* in CaP, providing a strong rationale for the biomarker and therapeutic utility of *ERG* splice variants, along with *C-MYC*.

## INTRODUCTION

Genetic rearrangements involving DNA sequences from different chromosomes or intrachromosomal regions have been extensively documented in various cancers, including prostate cancer (CaP) [1]. It has been reported that male hormone dependent *ETS*-related transcription factors play causal roles in CaP, as a result of rearrangements. Hence, gene fusions involving the *ETS* family have potential value in cancer diagnosis, prognosis, and therapy [2-5]. Of these, overexpression of the *ETS*-related gene (*ERG*) [6, 7], resulting from the fusion of *ERG* coding sequences to the androgen-responsive *TMPRSS2* gene [8], represents the most common subtype among *ETS* fusions, with a prevalence of approximately 50% in clinically localized prostate cancers [1-4, 9-12]. In addition, studies evaluating the expression of *ERG* in epithelial cells of matched benign and malignant prostate cells from a large patient cohort indicate that CaP cells harboring *TMPRSS2-ERG* fusion show overexpression of *ERG* in 60-70% of patients [10]. This genomic rearrangement is now established as one of the most common mechanisms of oncogenic activation in CaP [3-5]. *ERG* has also been originally implicated in Ewing's sarcoma and acute myeloid leukemia [13-15].

The multi-exon structure of *ERG* is transcribed to nine different splice variants by a combination of alternative transcription initiation, mRNA splicing, and transcription termination [6, 7, 16]. These variants can be divided into near full length (lacking 32 N-terminal amino acids) *Type I ERG*, containing the DNA-binding domain (DBD), with the embedded nuclear localization signal (NLS) and *Type II ERG*, a truncated form lacking DBD/NLS coding sequences [17]. The analysis of CaP associated fusion transcripts has revealed the presence of multiple splice variants, potentially exhibiting different biological activities and correlating with different tumor phenotypes [12, 13, 17-22]. Along these lines, our laboratory cloned and sequenced the relatively abundant full length *TMPRSS2-ERG* cDNAs from a pool of mRNAs from six *TMPRSS2-ERG* positive prostate tumors. In addition to the expected near full length fusion transcripts, we also identified mRNA splice variants of fusion transcripts lacking the C- terminus of *ERG* beyond exon 12 (Owczarek nomenclature), which was replaced by sequences resulting in the addition of 4 and 70 unique amino acids in TEPC1 and ERG8, respectively [16, 23]. The translated products of most of the various *ERG* transcripts have been shown to function as oncoproteins retaining the *ETS* domain with transforming activity [6, 14, 24, 25]. In addition, *ERG*, similar to other members of the *ETS* family, has been described as a mediator of mitogenic signals, such as mitogen activator protein kinases [26].

The relationship between the *ERG* transcripts and prognosis of CaP, however, is not clear. Analyses have shown that a specific variant is associated with an aggressive form of disease [27], while Hermans et al. [9] showed a favorable prognosis of CaP with another variant. This scenario highlights the need to evaluate the specific function of the different *ERG*-encoded proteins in the context of CaP. This knowledge will further our understanding of *ERG* towards its clinical utility, including patient stratification, treatment monitoring, and therapeutic targeting of CaP. In this regard, we hypothesized that the variants may function either additively, synergistically, or in a dominant negative fashion due to potential interactions, or competition, between the different splice forms. Such an interaction may modify the physiological responses of ERG protein, which include transcriptional activation, cell growth, differentiation, and apoptosis. To address this, we utilized the two most abundant prototypic variants described in CaP tumors, designated *TMPRSS2-ERG3* (*Type I*) and *TMPRSS2-ERG8* (*Type II*), and assessed their effects alone and in combination, using *in vitro* cell culture models. Our data show that *TMPRSS2-ERG8* exhibited a dominant negative effect over *TMPRSS-ERG3* mediated transcriptional regulation. Furthermore, the demonstration of a physical interaction between the variants may provide the basis for the functional antagonism. In addition, analysis of the ratio of *Type I/Type II ERG* in relation with the prevalent oncogene *C-MYC* may be of value as a biomarker in prostate cancer diagnosis and prognosis.

## RESULTS

### ERG encoded proteins localize to distinct regions within cells

**Figure 1:**
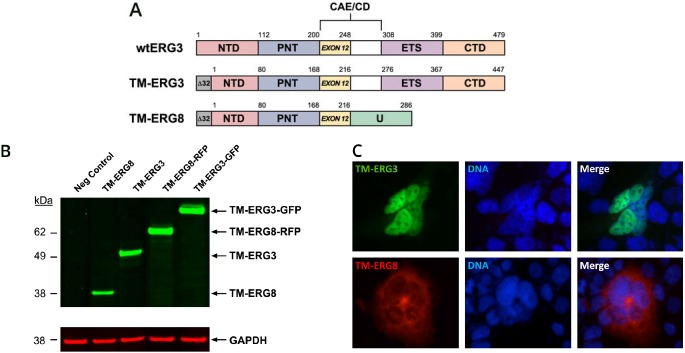
*TMPRSS2-ERG* splice variants and subcellular localization of the respective proteins (A) Schematic representation of TM-ERG3, TM-ERG8 and wild type ERG3 proteins. NTD, N- terminal domain; PNT, Pointed domain; CAE, Central alternative exons; CD, Central domain; ETS, DNA binding domain; CTD, C-terminal domain; U, Unique 70 amino acids (B) Expression of TM-ERG3 and TM-ERG8 proteins in HEK293 cells. Cell lysate, separated on a SDS-PAGE gel and transferred to PVDF membrane, was probed with ERG specific 9FY monoclonal antibody. TM-ERG3 and TM-ERG3-GFP correspond to 53 and 79 kDa proteins, respectively. TM-ERG8 and TM-ERG8-RFP correspond to 38 and 64 kDa proteins, respectively. Glyceraldehyde 3- phosphate dehydrogenase, GAPDH, was used as an internal control. (C) Subcellular localization of TM-ERG3-GFP and TM-ERG-RFP proteins in HEK293 cells. ERG3 was localized to the nucleus while ERG8 was found predominantly in the cytoplasm. DAPI was used for staining the nuclei.

For functional studies, *TMPRSS2-ERG3* and *TMPRSS2-ERG8* coding sequences were cloned into a pIRES-EGFP eukaryotic expression vector. A schematic representation of both constructs, in comparison to wild type *ERG3 (wt-ERG3)*, is presented in Figure 1a. In order to examine the subcellular localization of ERG proteins, *TMPRSS2-ERG3* and *TMPRSS2-ERG8* coding sequences were fused to GFP and RFP tags at the C-termini, respectively. The expression of tagged proteins was verified in HEK293 cells by immunoblot analysis, using the CPDR ERG mouse monoclonal antibody, 9FY [5, 28]. Since 9FY recognizes an epitope present at the N-terminus, both *TMPRSS2-ERG3* and *TMPRSS2-ERG8* can be detected in the molecular weight range of 53 kDa and 38 kDa, respectively. Alternatively, TMPRSS2-ERG3-GFP and TMPRSS2-ERG8-RFP revealed chimeric proteins in the range of 79 and 64 kDa, respectively (Figure 1b). The subcellular localization of proteins was examined by microscopy, upon transfection of *TMPRSS2-ERG3-GFP* and *TMPRSS2-ERG8-RFP* vectors in HEK293 cells. The backbone plasmid vector was transfected into cells as a negative control. As expected, ERG3, which contains the DBD and NLS, was localized to the nuclear compartment of cells. In contrast, the NLS-lacking ERG8 protein was detected in the cytoplasm (Figure 1c). The results demonstrate that the majority of ERG3 and ERG8 proteins exhibit distinct subcellular localizations in cells.

### ERG8 interferes with the transcriptional regulatory function of ERG3

**Figure 2:**
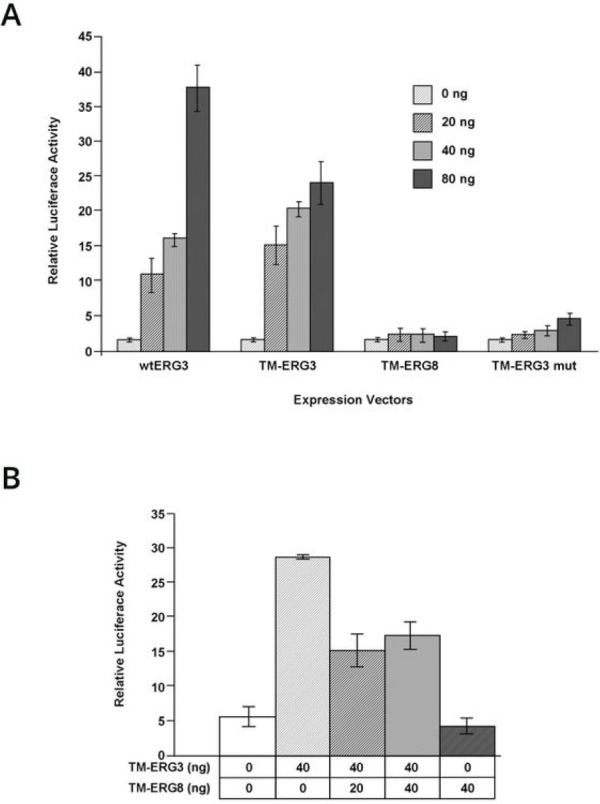
Transcriptional activation of downstream targets by *TMPRSS2-ERG* splice variants (A) Modulation of expression of a luciferase reporter construct containing *mef2c* enhancer. The reporter construct was co-transfected with 0, 20, 40 and 80 ng of vectors expressing *wtERG3*, *TM-ERG3*, *TM-ERG8* or *TM-ERG3* mutant. Each assay was repeated a minimum of 3 times. The data from a representative experiment is presented here. The extent of activation is presented in the form of relative luciferase activity, which increased with increasing concentrations of vector for both *wtERG3* and *TM-ERG3*. *TM-ERG8* and *TM-ERG3mut* resulted in baseline levels of activity. (B) Transdominant effect of *TM-ERG8* on transcriptional activation mediated by *TM- ERG3*. A fixed amount of luciferase reporter (40 ng) and *TM-ERG3* (40 ng) were co-transfected along with an increasing amount of *TM-ERG8* (0-40 ng) into HEK293 cells. A decrease in relative luciferase activity was observed with increased *TM-ERG8*.

The effect of ERG on downstream regulatory processes was examined using a reporter construct in which the mouse myocyte enhancer factor 2 (*mef2c*) sequence was cloned upstream of the luciferase gene. Cells were transfected with the luciferase construct alone and also in combination with *wt-ERG3*, *TMPRSS2-ERG3*, or *TMPRSS2-ERG8*. A mutant *TMPRSS2-ERG3* construct, containing an inactivating N-terminal +1 frame shift mutation, served as a negative control. While cells transfected with reporter construct by itself showed baseline luciferase activity, co-transfection with either the *wtERG3* or *TMPRSS2-ERG3* activated the transcription mediated by the *mef2c* reporter. Transfection with either *TMPRSS2-ERG8* or *mutTMPRSS2-ERG3*, on the other hand, did not lead to an increase of luciferase activity (Figure 2a). Considering the presence of multiple *TMPRSS2-ERG* transcriptional variants in human prostate tumors, we also evaluated the effect of *TMPRSS2-ERG8* on *TMPRSS2-ERG3* function. For this purpose, the expression constructs were co-transfected along with the luciferase reporter construct such that *TMPRSS2- ERG3* was kept constant, while *TMPRSS2-ERG8* was transfected in increasing concentrations, in HEK293 cells. The results showed a loss of the transcriptional activator function of *TMPRSS2- ERG3* with the addition of *TMPRSS2-ERG8*. The addition of either 20 ng or 40 ng of the *TMPRSS2-ERG8* expression plasmid in the co-transfection assay exhibited around 50% reduction in luciferase activity in comparison to TM-ERG3 alone (Figure 2b). This suggests that there is an interference of *TMPRSS2-ERG3* mediated activation by *TMPRSS2-ERG8*.

### Ectopic expression of ERG8 in VCaP cells influences the endogenous level of ERG3

**Figure 3:**
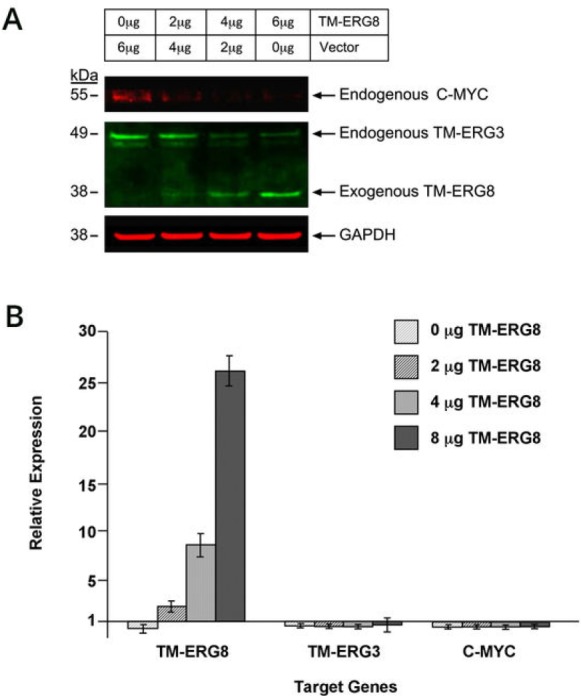
Effect of ectopic expression of TMPRSS2-ERG8 in prostate cancer derived VCaP cells (A) Ectopic expression of *TM-ERG8* affects the level of endogenous TM-ERG3 and C- MYC proteins in VCaP cells. Cells were transfected with different amounts of *TM-ERG8* as indicated, lysed, and analyzed for protein expression of TM-ERG3 and C-MYC. Signals detected were quantitated using Odyssey software (data not shown). A decrease in endogenous TM- ERG3 protein and C-MYC protein was evident with the expression of TM-ERG8. The experiment was repeated three times. (B) Analysis of the effect of *TM-ERG8* on endogenous *TM-ERG3* and *C-MYC* at the RNA level. Quantitative RT-PCR was performed using RNA from VCaP cells transfected with *TM-ERG8*. Both *TM-ERG3* and *C-MYC* remained at baseline levels of expression.

Next, we analyzed the effect of *TMPRSS2-ERG8* expression in VCaP cells, which are known to harbor fusions involving *ERG* and *TMPRSS2* genes. VCaP cells were transfected with the *TMPRSS2- ERG8* vector in increasing concentrations (0, 2, 4, 6 μg), along with an empty vector in order to maintain the quantity of transfected plasmid DNA constant in each sample. Results revealed that as ectopically expressed ERG8 protein levels were increased, protein level of endogenous ERG3 was decreased, as shown in immunoblot assays (Figure 3a). Earlier studies from our laboratory showed that ERG3 binds to the *C-MYC* P2 promoter downstream elements and activates the expression of *C-MYC* [29]. Based on this, we evaluated the effect of ERG8 on C-MYC expression in VCaP cells. The results showed that C-MYC protein level also exhibited a decreasing trend similar to ERG3 protein level. The quantitation of signals in the immunoblot by Odyssey software, revealed the following: i) There was an increase in TMPRSS2-ERG8 expression corresponding to the amount of DNA transfected; ii) The endogenous TMPRSS2- ERG3 registered a 32% decrease in protein level in cells transfected with 6 μg of TMPRSS2- ERG8 DNA.; iii) The endogenous C-MYC showed a trend similar (38% decrease) to that of endogenous TMPRSS2-ERG3. Furthermore, the analysis at the RNA level by qPCR showed that both *TMPRSS2-ERG3* and *C-MYC* remained unchanged, suggesting the modulation by *TMPRSS2-ERG8* is at the protein level (Figure 3b).

### Bimolecular fluorescence complementation assay shows interaction between splice variants

**Figure 4:**
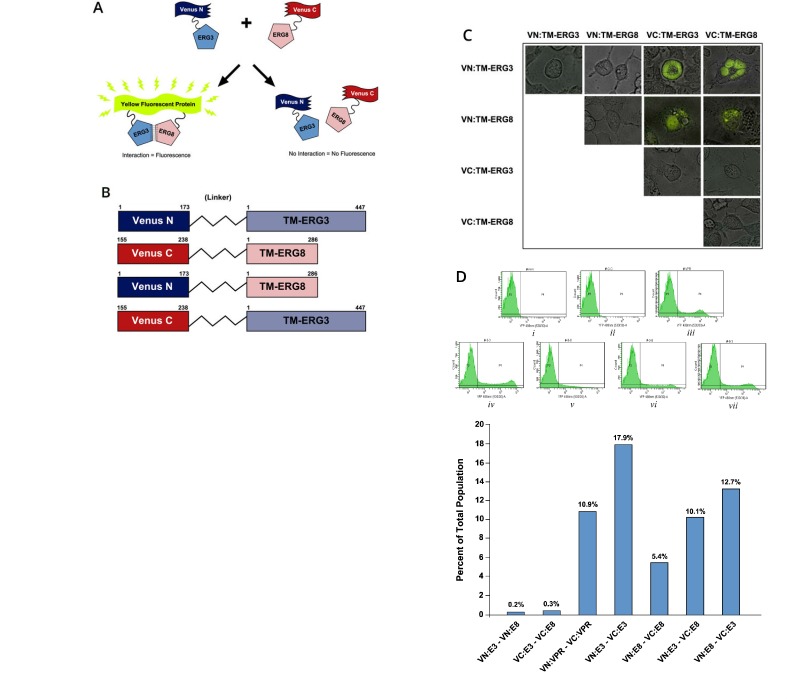
Analysis of the interaction between *TMPRSS2-ERG3* and *TMPRSS2-ERG8* by BiFC assay (A) Schematic representation of the bimolecular fluorescence complementation assay. (B) Schematic representation of the chimeric proteins containing N- or C-terminal regions of the *Venus* gene and coding sequences of *TM-ERG* splice variants. (C) Demonstration of the interaction between TM-ERG3 and TM-ERG8 proteins in live HEK293 cells. Yellow fluorescence is visible upon reconstitution of the Venus protein, resulting from the interaction of splice variants. Cells were visualized by microscopy using Leica, DMIRE2. (D) Quantitation of the fluorescence was performed by flow cytometry: *i*, Venus N-terminal constructs transfected together (negative control); *ii*, Venus C-terminal constructs transfected together (negative control); *iii*, *VN*- and *VC*- constructs for Vpr transfected together (positive control); *iv*, *VN*- and *VC*- constructs for *TM-ERG3* transfected together; *v*. *VN*- and *VC*- constructs for *TM- ERG8* transfected together; *vi*, *VN:TM-ERG3* and *VC:TM-ERG8* constructs transfected together; *vii*, *VN:TM-ERG8* and *VC:TM-ERG3* constructs transfected together; *viii*, Graphical representation of flow cytometry results.

To address the underlying basis for the interference of *TMPRSS2-ERG3* mediated functions by *TMPRSS2-ERG8*, we assessed the potential interaction between ERG3 and ERG8 proteins through fluorescence microscopy and flow cytometry using the bimolecular fluorescence complementation (BiFC) assay in live cells. This assay involves a chimeric Venus protein which emits fluorescence upon dimerization in live cells (Figure 4a). For this purpose, we generated constructs containing *TMPRSS2-ERG3* and *TMPRSS2-ERG8* coding sequences fused to either N- or C-terminal coding sequences of the Venus reporter (Figure 4b). HIV-1 *Vpr* constructs were used as positive controls, as *Vpr* has been well characterized and shown to oligomerize [30]. As expected, we observed that *VN-Vpr* and *VC-Vpr* constructs co-transfected in HEK293 cells produced fluorescent signal, which was found to be localized in the nuclear region. In addition, co-transfection of *VN-ERG3* and *VC-ERG3* resulted in fluorescence, also observed in the nucleus. Similar results were seen for *VN-ERG8* and *VC-ERG8* co-transfection in which fluorescence was observed in the cytoplasm. Furthermore, co-transfection of *VN-ERG3* and *VC-ERG8*, as well as *VC-ERG3* and *VN-ERG8*, resulted in fluorescent protein in the nucleus, indicating a direct interaction between protein products of the two splice variants (Figure 4c). The transfection of two *VN*- constructs or the two *VC*- constructs together, as negative controls, showed no fluorescence in cells. In addition, a *VN*- or *VC*- construct transfected by itself also resulted in no fluorescence. The results from transfected cells independently analyzed by flow cytometry were consistent with the results obtained by microscopy (Figure 4d). The *Vpr* positive control cell population contained 10.9% cells positive for fluorescence (Figure 4d-*iii*). The positive control for ERG3 resulted in 17.9% positive cells (Figure 4d-*iv*), and for ERG8 resulted in 5.4% positive cells (Figure 4d-*v*). As predicted, co-transfection of *VN-ERG3* and *VC-ERG8*, as well as *VC-ERG3* and *VN-ERG8*, showed 10.1% and 12.7% cells positive for fluorescence, respectively (Figure 4d-*vi* and *vii*). Negative controls displayed ≤ 0.3% base line positivity (Figure 4d-*i* and *ii*). Thus, BiFC assay demonstrated interaction between ERG3 and ERG8 variants in live cells.

### Clinicopathological analysis of patient data reveals a correlation between C-MYC and ERG3/ERG8 ratios

The intriguing observation that altered *ERG* expression can significantly affect *C-MYC* levels in the VCaP cells prompted us to re-examine *Type I/Type II* ratios and *C- MYC* expression from our previously published qRT-PCR data from laser capture micro- dissected (LCM) prostate cancer cells [10, 17]. Our previous report showed that increased *Type I* (*ERG3)/Type II* (*ERG8)* ratio is associated with a higher Gleason sum and poorly differentiated phenotype. In contrast, a decrease in *Type I/Type II ERG* ratio was associated with favorable clinical-pathologic data [17]. Consistent with these observations, we also noted a significant correlation between *Type I/Type II ERG* ratio and *C-MYC* mRNA levels (rho = 0.37, P = 0.013) within the same specimens (Table 1). In comparison, we also analyzed the relationship between *PCA3* expression levels (a gene up-regulated in CaP patients, though not an ERG transcriptional target) and *Type I/Type II ERG* ratio, which did not show any correlation (rho = -0.06, P = 0.682; Table 1). We then compared the differences in ratios of *Type I/Type II ERG* across quartile groups of *C-MYC* gene expression, and found a trend in which groups with higher levels of *C- MYC* expression had higher *Type I/Type II ERG* ratios, (P = 0.0894). PCA3 analysis across quartile groups again revealed that higher gene expression levels did not correlate to higher *Type I/Type II ERG* ratio (P = 0.842). Further, from a biological standpoint, we compared the T*ype I/Type II ERG* ratio between down-regulated *C-MYC* (<0.5 fold) vs. up-regulated *C-MYC* (>2 fold*)*. This analysis showed that up-regulated *C-MYC* had higher *Type I/Type II ERG* ratio compared to down-regulated *C-MYC* which shifted even closer towards significance (P = 0.056; Table 2).

**Table 1:** Spearman's correlation analysis of CaP oncogenes with *Type I/Type II ERG* ratio

Gene Expression	*Type I/Type II* ratio			
	*N*	rho	P
*C-MYC* fold difference	45	0.37	*0.013
*PCA3* fold difference	51	-0.06	0.682

* **P < 0.05 was considered statistically significant.**

Spearman's correlation analysis reveals that increased C-MYC expression correlates with higher Type I/II ERG ratios. This correlation is not noted for analysis with PCA3

**Table 2:** Correlation of *C-MYC* gene expression with *Type I/Type II ERG* ratio

Biological Breakdown	*Type I/Type II* ratio		
	*N*	Median	P
*C-MYC* fold difference:	(45)	0.37	0.063
			**0.056
< 0.5 fold (down-regulation)	8	0.28	**
0.5-2 fold (relatively unchanged)	23	0.46	
> 2.0 fold (up-regulation)	14	0.72	

** **Trending towards significance. P < 0.05 was considered significant.**

As C-MYC gene expression is found to be down-regulated in CaP patients, Type I/II ERG ratio is low. When C-MYC gene expression is up-regulated in CaP patients, Type I/II ERG ratio is found to be high.

## DISCUSSION

Genomic rearrangements of oncogenes are established features in several human cancers and have been recognized for decades [1, 3, 5]. The involvement of fusion genes in prostate cancer has been defined by Tomlins et al. [4] and consistently reported by others [3, 5, 17-19, 23]. The fusion between androgen regulated *TMPRSS2* and *ERG* genes is present in ~50% of prostate cancer patients in Western countries [3, 4, 17, 29, 31]. Analysis of fusion gene transcripts from prostate cancer patients by several investigators has shown the presence of multiple forms containing varying lengths of *TMPRSS2* (promoter and exon 1 and 2) and *ERG*. The predominant form noted involves the fusion of *TMPRSS2* exon 1 and *ERG* exon 4 (Tomlins nomenclature)/exon 8 (Owczarek nomenclature) [4, 16, 23]. Recent studies have shown that *TMPRSS2-ERG* expression is associated with an aggressive CaP phenotype [18, 32] while others noted that splice variants exhibit varied level of transforming activity [12]. Interestingly, the splice variants have been suggested to play both a positive and negative role in the prognosis of CaP [10, 33]. In addition, Wang et al. showed that the presence of the various isoforms led to increased proliferation/invasion, while knockdown led to decreased ectopic tumor size [19]. However, the functional significance of the individual transcripts, as well as their effect on each other, was not evaluated. Here, we have characterized the most prevalent prototypic fusion transcripts (*TMPRSS2-ERG3* and *TMPRSS2-ERG8)* that were originally identified in our previous report [17].

As a prelude to functional evaluation, we initially characterized the synthesis and subcellular localization of the proteins encoded by *TMPRSS2-ERG3* and *TMPRSS2-ERG8*. The proteins exhibited distinct subcellular localization; ERG3 localized predominantly in the nucleus and ERG8 exhibited cytoplasmic distribution. These distinct biological properties substantiated that *TMPRSS2-ERG3* and *TMPRSS2-ERG8* were appropriate fusion variants for investigating the functional significance of splice variants, as well as examining their influence on one another.

The transcriptional regulatory function of proteins encoded by these splice variants was explored through use of the enhancer from the mouse *mef2c* gene which has been shown to be active in the vascular endothelium during embryogenesis [34] and in adulthood where endogenous ERG protein is highly expressed [21, 35, 36]. We utilized the *mef2c* vascular endothelial enhancer sequence, containing a cluster of four conserved binding sites for *ETS* factors, linked to the luciferase reporter, to assess the transcriptional regulatory function of *ERG* splice variants. Both full length (*wtERG3*) and the N-terminus truncated *Type I ERG* protein (*TMPRSS2-ERG3*) activated the transcription from the *mef2c* driven luciferase reporter construct in HEK293 cells. Consistent with the absence of the NLS in the *Type II ERG* product, the ERG8 protein did not alter the basal promoter activity of the reporter. However, co-expression of *TMPRSS2-ERG3* and increasing concentrations of *TMPRSS2-ERG8* expression vectors showed an inhibition of *TMPRSS2-ERG3*-mediated activation of the *mef2c* reporter. The results suggested that TMPRSS2-ERG exhibited a dominant negative effect on the function mediated by TMPRSS2-ERG.

Based on these findings, we then used VCaP cells which express both *TMPRSS2-ERG* variants, allowing us to carry out studies mimicking the *in vivo* context of prostate tumors. Specifically, these cells express high levels of *TMPRSS2-ERG8* (copy number) in comparison to *TMPRSS2-ERG3* and also respond to androgens. Hence, introduction of exogenous TMPRSS2- ERG8 into VCaP cells enabled us to assess its effect on the endogenous ERG3 level. An increase in expression of ERG8 resulted in a decrease in endogenous ERG3 protein in VCaP cells. Remarkably, C-MYC protein levels mirrored the decrease in ERG3 protein levels. Based on this, we suggest that ERG3, a transcriptional activator of *C-MYC*, is concomitantly regulated by ERG8. Interestingly, while an alteration at the protein level was observed with the addition of TMPRSS2-ERG8, such an effect was not evident at the RNA level. It should be noted that ERG8 lacks the DBD and embedded NLS, but retains the SAM-pointed and protein-protein interaction domain. Therefore, it is suggested that elevated levels of ERG8 likely result in a dominant negative effect due to dimerization-mediated quenching of functional ERG3. This was alluded to in the analysis performed by Zammarchi et al. [12], though not explored further as to biological function.

In order to further investigate the mechanistic basis of the transdominant effect between splice variants, it was important to elucidate whether there is a direct interaction between the two isoforms. The BiFC assay, utilizing a chimeric Venus protein, allowed us to examine this directly in live cells through fluorescence microscopy. Venus constructs that contained either the N-terminus or C-terminus coding sequences of the Venus protein, linked to either *TMPRSS2- ERG3* or *TMPRSS2-ERG8* coding sequences were first evaluated, as they are known to homodimerize [33, 37]. As expected, cells were found to fluoresce, either in the nuclear region, as in the case with *TMPRSS2-ERG3* or in the cytoplasmic region, as in the case with *TMPRSS2- ERG8* alone. The extent of fluorescence positive cells upon expression of chimeric TMPRSS2- ERG3 and TMPRSS2-ERG8 supports the presence of interaction domains at the N- and C- termini of ERG3 protein. When cells were co-transfected with both splice variants together, we again observed fluorescence in cells, indicating heterodimerization between the two splice variants in live cells. It is interesting to note, that while the subcellular localization of *Type I* and *Type II ERG* was found to differ, heterodimers containing ERG3 and ERG8 were observed within the nuclear compartment of cells. This suggests that protein-protein interactions are likely to take place upon synthesis in the cytoplasm, after which they are transported to the nucleus. As ERG8 does not contain the NLS, it is unlikely to be transported to the nucleus on its own.

Based on this observation, we envision a scenario in which both homodimers and heterodimers may exist. Thus, it is reasonable to expect that in the context of an altered ratio containing high *TMPRSS2-ERG8* over *TMPRSS2-ERG3*, there is a likelihood that heterodimers may predominate over the homodimers of ERG3. This possibility will lead to a diminished function of the *TMPRSS2-ERG3* variant. Thus, our experiments involving co-transfection of plasmids expressing *TMPRSS2-ERG3* and *TMPRSS2-ERG8* variants along with *mef2c* driven luciferase reporter construct showed a diminished activation, in comparison to cells transfected with *TMPRSS2-ERG3* alone. There is a possibility that heterodimers containing *TMPRSS2- ERG3* and *TMPRSS2-ERG8* may preferentially be subjected to degradation resulting in a decreased level of *TMPRSS2-ERG3* in VCaP cells. However, further investigation of this is necessary.

It has been shown that *C-MYC* amplification increases as carcinogenesis progresses and it is strongly associated with higher histopathological grades and Gleason's scores as well as with earlier disease progression and earlier cancer death [27, 38]. *C-MYC* is also a known downstream target of *TMPRSS2-ERG3* [23, 26, 29, 39, 40], however its relationship to *Type I/II ERG* ratio has not yet been examined. We therefore explored clinical patient data specifically for the direct relation of *Type I/II ERG* ratio and *C-MYC* gene expression levels. Indeed, it was observed that when oncogenic *C-MYC* was found to be up-regulated, the *Type I/II ERG* ratio was also high, corroborating with earlier data in which a high Gleason sum and poor overall patient prognosis were associated with an increased *Type I/II ERG* ratio. *C-MYC* levels would also be increased in this scenario, which could be involved in progression of the disease state. The results of this analysis represent a case for the use of the ratio of *Type I/Type II ERG,* in conjunction with *C-MYC* gene expression levels, to serve as potential prognostic markers for treatment of prostate cancer patients.

**Figure 5:**
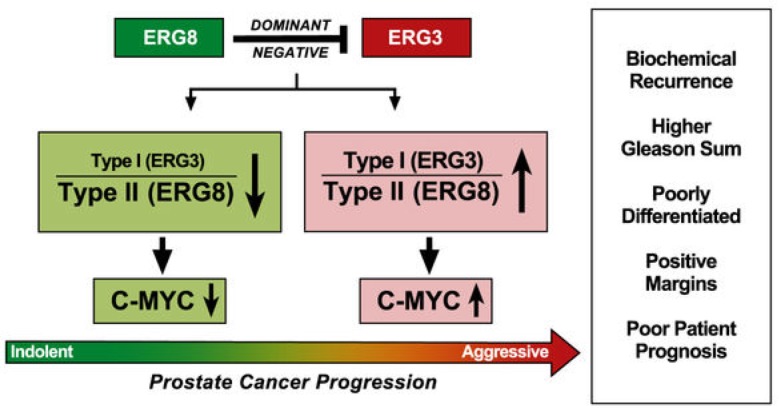
Mechanistic model of TM-ERG splice variants As levels of *TM-ERG8* are increased, the overall ratio of *Type I/Type II ERG* is effectively decreased, which is associated with lower levels of *C-MYC* and a more favorable patient prognosis. When levels of *TM-ERG8* are lower, the *Type I/II* ratio is effectively higher, which is found to be associated with higher levels of *C- MYC* and a more aggressive form of disease in patients.

Based on these findings we propose a mechanistic model in which *Type II ERG* (*TMPRESS2-ERG8*) regulates the activity of *Type I ERG* (*TMPRSS2-ERG3*), in a dominant negative manner (Figure 5). As *TMPRSS2-ERG8* is increased, *TMPRSS2-ERG3* is decreased and the downstream events regulated by *TMPRSS2-ERG3* are also likely inhibited. This scenario is also supported by the data on *C-MYC*, which has been shown as a regulatory target of *TMPRSS2- ERG3*. In accordance with this, an increased *TMPRSS2-ERG8* correlates with a lower level of both *TMPRSS2-ERG3* and *C-MYC*.

Overall, studies presented here show that the interaction between *Type I* and *Type II* splice variants of *TMPRSS2-ERG* present in prostate tumor tissues may lead to functional antagonism of selected variants. We hypothesize that an altered ratio of high *TMPRSS2-ERG8* in comparison to *TMPRSS2-ERG3* may be beneficial to prostate cancer patients. The results further highlight the potential for the ratio of *ERG* splice variants in combination with *C-MYC* expression to be exploited for diagnostic and therapeutic applications against prostate cancer.

## METHODS

### Cell culture

The Vertebral-Cancer of the Prostate (VCaP) cells and human embryonic kidney (HEK) 293 cells were obtained from American Type Culture Collection (ATCC; Manasses, VA, USA). VCaP and HEK293 cells were cultured in Dulbecco's Modified Eagle Medium (DMEM; ATCC) supplemented with 10% fetal bovine serum (FBS; ATCC). All cells were cultured in humidified conditions, at 37°C with 5% CO2. Media changes occurred every other day, and cells were passaged as needed, when confluent.

### Generation of expression vectors

*TMPRSS2-ERG3*, *TMPRSS2-ERG8*, and *wt-ERG3* (Flag- tagged) constructs were cloned into pIRES-EGFP plasmid vector, as previously described [17]. In addition, we generated *TMPRSS2-ERG3-GFP* and *TMPRSS2-ERG8-RFP* constructs by cloning *TMPRSS2-ERG3* and *TMPRSS2-ERG8* sequences into pAcGFP1-N1 and pDsRed- Monomer-N1 plasmid vectors, respectively. The expression of proteins was verified in HEK293 cells. Cell lysates derived from transfected cells were detected by western blot using 9FY ERG MAb. For the BiFC assay, sequences encoding the N-terminus (residues 1 to 173, VN) or C- terminus (residues 155 to 238, VC) fragments of the Venus fluorescence protein were fused to *TMPRSS2-ERG3* or *TMPRSS2-ERG8* coding sequences via a 10 amino acid linker. Plasmids were generated in pcDNA3 through a commercial vendor (GenScript; Piscataway, NJ, USA).

### Western blot

Cell pellets from transfection procedures were lysed in Mammalian Protein Extract Reagent (M-PER; Pierce/Thermo Scientific, Rockford, IL, USA). Following pre-cleaning by centrifugation, protein concentrations of cell lysates were determined by using Protein Assay Reagent (Bio-Rad, Hercules, CA, USA). Lysates equivalent to 25 μg of protein were separated on NuPAGE Bis-Tris (4-12%) gels (Invitrogen, Carlsbad, CA, USA) and transferred onto PVDF membranes. Membranes were blocked in Blocking Buffer (LI-COR, Lincoln, NE, USA) and incubated with specific antibodies against ERG splice variants (ERG MAb 9FY; Biocare Medical Inc., Concord, CA, USA), C-MYC (Epitomics, Burlingame, CA, USA) and GAPDH (Santa Cruz biotechnology, Santa Cruz, CA, USA). Membranes were washed in Tris-Buffered Saline + Tween 20 (TBST) before incubation with appropriate secondary antibodies (goat anti- Mouse IRDye 8000CW or goat anti-Rabbit IRDye 680CW, LI-COR). Signals of proteins detected were visualized and quantitatively measured using the Odyssey infra-red imaging scanner and software (LI-COR).

### Quantitative RT-PCR

RNA was isolated from cell pellets from transfection procedures using RNeasy RNA Isolation Kit (Qiagen; Germantown, MD, USA). cDNA was reverse transcribed from RNA, and quantitative RT-PCR was performed using 500 pg of cDNA. The appropriate primers for ERG3, ERG8, and C-MYC (Table 3) were used with gene-specific TaqMan probes for quantitative evaluation of ERG3 (5'- FAM-ACTAGGCCAGATTTACCA - 3') and C-MYC (5'- FAM-ACCTTTTGCCAGGAGCCTGCCTCT - 3') and SYBR Green for quantitative evaluation of ERG8, in 96-well plates. Glyceraldehyde 3-phosphate dehydrogenase (GAPDH) was used as an internal control gene. Results were calculated as a function of 2(−ΔΔCt).

**Table 3:** Primers used for analysis of RNA

Gene		Primer Sequences	Product	RefSeq
ERG3	Forward Reverse	5'-CAGTATATCCTGAAGCTACGCAAAGA-3' 5'-GGTCCAGGCTGATCTCCT-3'	80	NM182918.3
ERG8	Forward Reverse	5'-GGTACGAAAACACCCCTGTG-3' 5'-CCAAATCAACAGAGGCAGAA-3'	150	AY204742.1
C-MYC	Forward Reverse	5'-ACCACCAGCAGCGACTCTGA-3' 5'-TCCAGCAGAAGGTGATCCAGACT-3'	117	NM002467.4
GAPDH	Forward Reverse	5'-GAGCCACATCGCTCAGACACC-3' 5'-GTAGTTGAGGTCAATGAAGGGGTC-3'	147	NM001289745.1

Forward and reverse primer sequences for *TM- ERG3, TM-ERG8, C-MYC* and *GAPDH* used in quantitative RT-PCR.

### Subcellular localization of ERG splice variants

HEK293 cells were seeded onto poly-lysine coated 12 mm round cover glass slides at 1×10^5^ cells per well in 12-well plates. Cells were co- transfected with 1 μg each *TMPRSS2-ERG3-GFP* and *TMPRSS2-ERG8-RFP* vectors using Lipofectamine 2000. After 72 hours, cells were washed with PBS, mounted in ProLongFade Gold (Invitrogen) anti-fade mounting reagent containing 4'6-diamidino-2-phenylindole (DAPI) for DNA staining. Cellular localization of ERG3-GFP and ERG8-RFP proteins were visualized by using an inverted Leica DMIRE2 microscope equipped with a QImaging Retiga-EX CCD camera (Surrey, BC, Canada), operated by OpenLab Software.

### Dual Luciferase Assays

HEK293 cells were transfected to examine the regulatory efficiency of both *TMPRSS2-ERG3* and *TMPRSS2-ERG8* on the *ETS*-regulated promoter of the myocyte- specific enhancer factor 2, *mef2c*. The endothelial cell enhancer derived *mef2c* was excised from pmef2c-F7-3-lacZ (a kind gift from Dr. Brian L. Black, UCSF) [34] and cloned upstream of a luciferase reporter in pGL4.24[luc2P/minP] in the *mef2c* construct. Cells seeded at 2.5×10^4^ cells per well in 48-well plates were co-transfected with the luciferase construct and either *wt-ERG3*, *TMPRSS2-ERG3*, *TMPRSS2-ERG8*, or a mutated *TMPRSS2-ERG3* construct, along with a Renilla construct at various doses, using Lipofectamine 2000 (Invitrogen). Cells were lysed *in situ* 24 hours post-transfection, rocked for 15 minutes at room temperature, and centrifuged at 15,000 × *g* for 15 minutes to pellet the cell debris. Cell supernatants were loaded onto Blackwell 96-well plates and evaluated for luciferase activity using the Dual Luciferase Reporter Assay System (Promega, Madison, WI, USA).

### Overexpression of TMPRSS2-ERG8 in VCaP cells

VCaP cells were transfected with *TMPRSS2- ERG8* (pIRES-TMPRSS2-ERG8-EGFP) vector in increasing concentrations using Lipofectamine 2000. Briefly, VCaP cells were seeded at 2×10^6^ cells in 100 mm plates. Cells were incubated overnight at 37°C to reach 50% confluency at the time of transfection. The next day, cells were transfected with *TMPRSS2-ERG8* (0, 2, 4, or 6 μg) using Lipofectamine 2000. An empty vector (pIRES-EGFP) was co-transfected into the cells, in order to maintain the amount of transfected DNA constant in each condition (6μg), where necessary. Cells were harvested 72 hours post-transfection using cell scrapers, centrifuged at 5,000 × *g* to pellet the cells, washed twice with PBS, and lysed for further analysis.

### Bimolecular Fluorescence Complementation Assay

HEK293 cells were seeded at 5×10^4^ cells in 35mm glass bottom dishes coated with poly-D-lysine. Cells were co-transfected with 1 μg each of *VN-ERG3* and *VC-ERG8* or *VC-ERG3* and *VN-ERG8* vectors using Lipofectamine 2000. VN- and VC- plasmids of the same splice variant transfected together were used as a positive control and both VN- or VC- plasmids transfected together were used as a negative control. After 24 hours, cells were washed once with PBS and stained with Hoechst 33342 nuclear stain (Invitrogen). Cells were again washed and visualized for presence of yellow fluorescent protein, indicating interactions between the splice variants, using microscopy (Leica, DMIRE2). For quantification of fluorescent cells using flow cytometry, HEK293 cells were seeded at 1×10^6^ cells in 100mm plates. Transfections were carried out as above. After 24 hours, cells were washed and stained with Hoechst 33342 nuclear stain. Cells were trypsinized, pelleted, washed, and resuspended in PBS + 2% FBS. The cell suspension was filtered into flow cytometry tubes. An unstained control and a Hoechst-only stained control were used to set up gates, and quantification was performed for each set of transfections.

### Clinicopathological analysis of patient data

The prostate tissue specimens used in this study were obtained from radical prostatectomy procedures under an Institutional Review Board– approved protocol at Walter Reed Army Medical Center. Detailed methods of laser capture microdissection (LCM) of tumor and benign epithelial cells, as well as quantitative gene expression are described in an earlier report from our laboratory [17]. Quantitative RT-PCR was carried out to analyze the expression of *Type I* and *Type II ERG* splice variants and *C-MYC* in LCM selected prostate cancer cells. We then examined the *Type I/Type II ERG* ratio in relation to *C-MYC* and *PCA3* gene expression levels through Spearman's correlation analysis. The differences of *Type I/Type II ERG* ratios across *C-MYC* and *PCA3* quartile groups, as well as down-regulated *C-MYC* vs. up-regulated *C-MYC*, were compared by using a Kruskal-Wallis test. A P-value of <0.05 was considered statistically significant.
